# Gait Characteristics and Fatigue Profiles When Standing on Surfaces with Different Hardness: Gait Analysis and Machine Learning Algorithms

**DOI:** 10.3390/biology10111083

**Published:** 2021-10-22

**Authors:** Zhenghui Lu, Dong Sun, Datao Xu, Xin Li, Julien S. Baker, Yaodong Gu

**Affiliations:** 1 Faculty of Sports Science, Ningbo University, Ningbo 315211, China; luzhenghui_nbu@foxmail.com (Z.L.); xudatao3@gmail.com (D.X.); lixin_nbu@foxmail.com (X.L.); 2 Savaria Institute of Technology, Eötvös Loránd University, 9700 Szombathely, Hungary; 3 Centre for Health and Exercise Science Research, Department of Sport and Physical Education, Hong Kong Baptist University, Hong Kong 999077, China; jsbaker@hkbu.edu.hk

**Keywords:** long time standing, floor mats, gait analysis, gender, fatigue, KNN classification algorithm

## Abstract

**Simple Summary:**

The purpose of this study was to explore if an anti-fatigue soft mat could improve the gait performance after standing for long periods and to examine if a machine-learning algorithm could evaluate fatigue state objectively. Compared with standing directly on the hard ground, using an anti-fatigue mat could reduce the negative effect of standing for a long time (4 h). The machine-learning algorithm demonstrated moderate accuracy in measuring fatigue. The accuracy of gait parameters used to consider a non-fatigued state following the use of an anti-fatigue mat was higher than that of the fatigue state. The results may indicate that it is beneficial to use anti-fatigue mats when standing for long periods, and it is feasible to use gait parameters and machine-learning algorithms to detect fatigue.

**Abstract:**

Background: Longtime standing may cause fatigue and discomfort in the lower extremities, leading to an increased risk of falls and related musculoskeletal diseases. Therefore, preventive interventions and fatigue detection are crucial. This study aims to explore whether anti-fatigue mats can improve gait parameters following long periods of standing and try to use machine learning algorithms to identify the fatigue states of standing workers objectively. Methods: Eighteen healthy young subjects were recruited to stand on anti-fatigue mats and hard ground to work 4 h, including 10 min rest. The portable gait analyzer collected walking speed, stride length, gait frequency, single support time/double support time, swing work, and leg fall intensity. A Paired sample t-test was used to compare the difference of gait parameters without standing intervention and standing on two different hardness planes for 4 h. An independent sample t-test was used to analyze the difference between males and females. The K-nearest neighbor (KNN) classification algorithm was performed, the subject’s gait characteristics were divided into non-fatigued and fatigue groups. The gait parameters selection and the error rate of fatigue detection were analyzed. Results: When gender differences were not considered, the intensity of leg falling after standing on the hard ground for 4 h was significantly lower than prior to the intervention (*p* < 0.05). When considering the gender, the stride length and leg falling strength of female subjects standing on the ground for 4 h were significantly lower than those before the intervention (*p* < 0.05), and the leg falling strength after standing on the mat for 4 h was significantly lower than that recorded before the standing intervention (*p* < 0.05). The leg falling strength of male subjects standing on the ground for 4 h was significantly lower than before the intervention (*p* < 0.05). After standing on the ground for 4 h, female subjects’ walking speed and stride length were significantly lower than those of male subjects (*p* < 0.05). In addition, the accuracy of testing gait parameters to predict fatigue was medium (75%). After standing on the mat was divided into fatigue, the correct rate was 38.9%, and when it was divided into the non-intervention state, the correct rate was 44.4%. Conclusion: The results show that the discomfort and fatigue caused by standing for 4 h could lead to the gait parameters variation, especially in females. The use of anti-fatigue mats may improve the negative influence caused by standing for a long period. The results of the KNN classification algorithm showed that gait parameters could be identified after fatigue, and the use of an anti-fatigue mat could improve the negative effect of standing for a long time. The accuracy of the prediction results in this study was moderate. For future studies, researchers need to optimize the algorithm and include more factors to improve the prediction accuracy.

## 1. Introduction

The Canadian Centre for Occupational Health and Safety pointed out in 2016 that standing for a long time without adequate rest may cause many health problems, such as low back pain and muscle fatigue [1]. When it takes more than 50% of the time to complete work-related tasks standing up, the worker is considered to be standing for a long time, resulting in potential injury [2,3,4]. Standing posture is very common for many occupations. Most of the time, manufacturing and service industries need to work in a standing position. These occupations include cashiers, assembly line workers, health care workers, barbers, etc. [4,5,6,7,8]. Due to static contractions of the waist and lower limbs, standing posture for long periods may lead to fatigue and discomfort, which may lead to discomfort, especially in the lower extremities [9,10,11,12,13]. Muscle fatigue may cause biomechanical changes of the lower extremities [14], and excessive fatigue can even cause oxidative stress [15] and musculoskeletal diseases [2]. The degree of general discomfort may increase with time, and people usually feel a change in the degree of discomfort in the first 30 and 90 min before standing, feet, ankles, back and hips are among many body areas affected [2,10,16,17]. After standing for 90 min, the subjects’ feet feel the most uncomfortable [18]. Studies by Gregory et al. have shown that waist discomfort may occur after standing for more than 2 h [11].

Not getting enough rest in a standing posture for a long time can lead to limited blood circulation and muscle discomfort, which eventually leads to fatigue [2,8]. Muscle fatigue is defined as loss of contractility caused by physical activity, inability to maintain expected strength, inability to work at expected exercise intensity, or reduced exercise performance during repeated or continuous stimulation [6]. Studies by Sartika et al. have shown that standing for a long time can lead to muscle fatigue in the lower back and legs, fatigue in the soleus muscle when standing for 5 min, and fatigue in the erector spine muscle at 30 min [10]. After standing, the waist and lower limbs remain static for a long time, leading to decreased muscle function, muscle fatigue, and discomfort in the legs [13,19]. Therefore, it is important to detect and warn of the fatigue and discomfort caused by standing.

In addition, it is worth noting that there is evidence that the proportion of men working standing up is higher than that of females [20]. Still, there are more reports of work-related musculoskeletal pain in females than in males [18,21,22]. This suggests that there may be gender differences in the effects of standing for a long time on the body. However, the study on the impact of gender on standing for a long time has not been fully explored.

Studies have shown that using supportive materials such as mats can reduce the discomfort and fatigue caused by standing for a long period [3,4,23,24]. The principle can be explained as adding a supporting interface between the foot and the ground, optimizing weight distribution [25]. In addition, some studies have shown that standing on a soft surface can cause muscles to produce small movements to improve blood flow and reduce fatigue and discomfort [4,24]. However, there is still some evidence that mating materials do not significantly improve fatigue and discomfort compared with hard floors [26,27]. Although there is some evidence that mating materials can reduce physical discomfort and fatigue, it does not reduce the physiological effects of muscle fatigue [5,26], such as changes in gait characteristics.

Gait is a behavioral feature of human walking, and normal walking requires the coordinated movement of many muscles and joints [28]. Studies have shown that lower limb muscle fatigue changes gait [29,30,31], such as the change of walking speed and stride length, etc. Gait analysis has been widely used to evaluate treatment and rehabilitation. Gait analysis can help identify abnormal gait and provide an objective reference for evaluating walking function and correcting abnormal gait [28,31,32,33,34]. Therefore, it is of great significance to accurately measure gait parameters. In the study of Amir Baghdadi et al. [35], subjects need to stack and transport several containers continuously, and the gait parameters are used to predict fatigue with an accuracy of 90%. In contrast, in this study, the effects of long-term static standing work on gait parameters were simulated to test whether intervention on different planes would get different results. To the best of our knowledge, no study has been published on the effect of standing for a long time on gait parameters.

In addition, some studies have shown that gait parameters, especially Spatio-temporal parameters (such as walking speed, stride length, and rhythm, etc.), are significantly correlated with fatigue [36,37,38,39], which means that they can be used as indicators of fatigue assessment. In this study, the KNN classification algorithm was used to design and detect gait parameters after standing for a long time.

In previous studies, gait analysis has been studied in the gait laboratory. Researchers used three-dimensional motion analysis systems and a dynamometer board to capture the subjects’ kinematic and dynamic data [28]. However, the laboratory cost is high, there are certain space technology requirements, clinical application is difficult [33]. Most importantly, individual or discontinuous gait data collected in the gait laboratory may not fully reflect the subjects’ gait characteristics [33]. Presently, portable wearable devices have been widely used in biomechanical analyses [40,41,42]. Therefore, in this study, we used a wearable intelligent analyzer (IDEEA, MiniSun, Fresno, CA, USA) to collect subjects’ gait data. The portable wearable device uses accelerometers and gyroscopes to record detailed data on types of body activity, duration, intensity, frequency, and gait parameters. Some studies have proved the reliability of IDEEA in measuring gait parameters [32,33,43,44,45,46].

Workers standing for long periods may lead to related occupational diseases. Therefore, it is important to carry out preventive interventions and properly detect fatigue and discomfort caused by long time standing. The purpose of this study was to explore whether anti-fatigue mats could improve the gait characteristics after standing for a long time and try to objectively detect the fatigue caused by standing for periods using the KNN classification algorithm.

## 2. Materials and Methods

### 2.1. Participants

Eighteen healthy young people (10 females and 8 males; 20–25 years old) participated in the study. All completed informed consent forms outlining experimental procedures and the purpose of the study. Ethical approval for the study was obtained from the university ethical committee. The subjects were healthy, without a history of lower back pain and lower limb injury. Table 1 summarizes the demographic information of the subjects related to age, height, and weight.

### 2.2. Equipments

**Figure 1:**
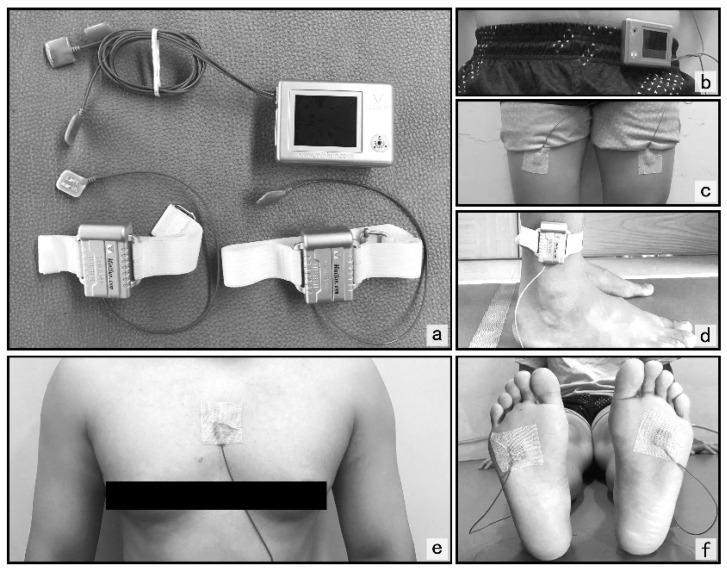
Outline of procedures. Schemes follow the same formatting. (**a**) Intelligent Device for Energy Expenditure and Activity (IDEEA); (**b**) main recorder position; (**c**) thigh sensor position; (**d**) secondary recorder’s position; (**e**) sternal sensor location; (**f**) foot sensor position.

Gait tests were performed using a wearable intelligent analyzer (IDEEA, MiniSun, Fresno, CA, USA) equipped with accelerometers and gyroscopes. The wearable intelligent analyzer consists of the main recorder and two secondary recorders. The primary recorder is worn at the waist, and the secondary recorder is placed directly above the outside of both ankles. The position of the sensor is that the sternum is perpendicular to the ground, the front of both thighs, and the proximal end of the fourth metatarsal head of both soles (Figure 1). The gait data are collected and transmitted to the main recorder by the sensor affixed to the subject. The IDEEA was easy to wear and had almost no interference on normal walking.

**Figure 2:**
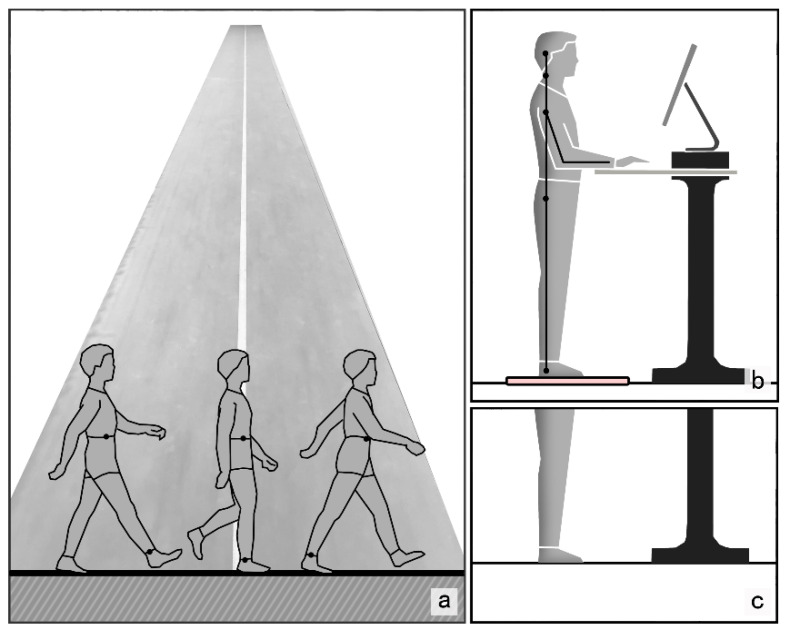
Schematic diagram of runway and subject standing. (**a**) Gait parameter acquisition track; (**b**) schematic diagram of the subjects standing on a mat; (**c**) schematic diagram of subjects standing on the floor.

The subjects were required to stand barefoot, and the mat material used in the experiment was a household PVC anti-fatigue mat, as shown in Figure 2.

### 2.3. Experimental Design

**Figure 3:**
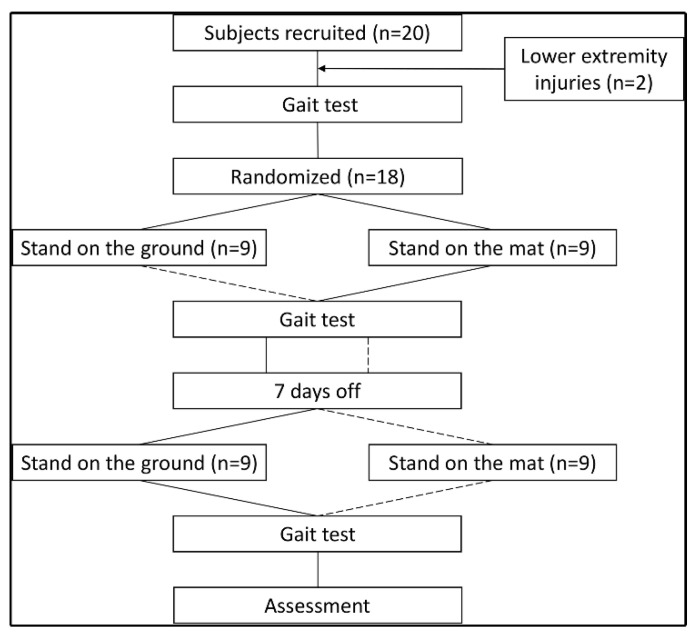
Flow chart.

The flow chart of the experiment is shown in Figure 3. The experiment was conducted on sunny afternoons to ensure similar relative humidity, between 13 and 17 o’clock, and the indoor temperature was fixed at 25–27 °C to prevent data contamination as a result of temperature change. The experiment simulated the conditions of standing and working. The subjects stood barefoot in front of a workbench with adjustable height and were free to choose to work, study, read or play games using a mobile phone or computer (Figure 2). Subjects were not allowed to support their body weight with their arms and were not allowed to tilt their center of gravity to one side of the body. A standing intervention was divided into two two-hour periods, providing a 10 min break between the two standing interventions, during which subjects could sit in a chair and rest. Each of the 18 subjects underwent either a mat stand or a floor stand, seven days apart to allow for adequate rest. The subjective feelings and physical discomfort of the subjects were collected during and after the experiment.

The next phase consisted of walking on a 10-m indoor track. Before the test, the subjects first wore IDEEA to walk freely on the track to familiarize themselves with the environment and equipment. After the start of the test, the subjects were asked to walk four times (40 m) on the track at a comfortable speed of their own choice, and the gait parameters were collected with IDEEA.

### 2.4. Outcome Measures

**Figure 4:**
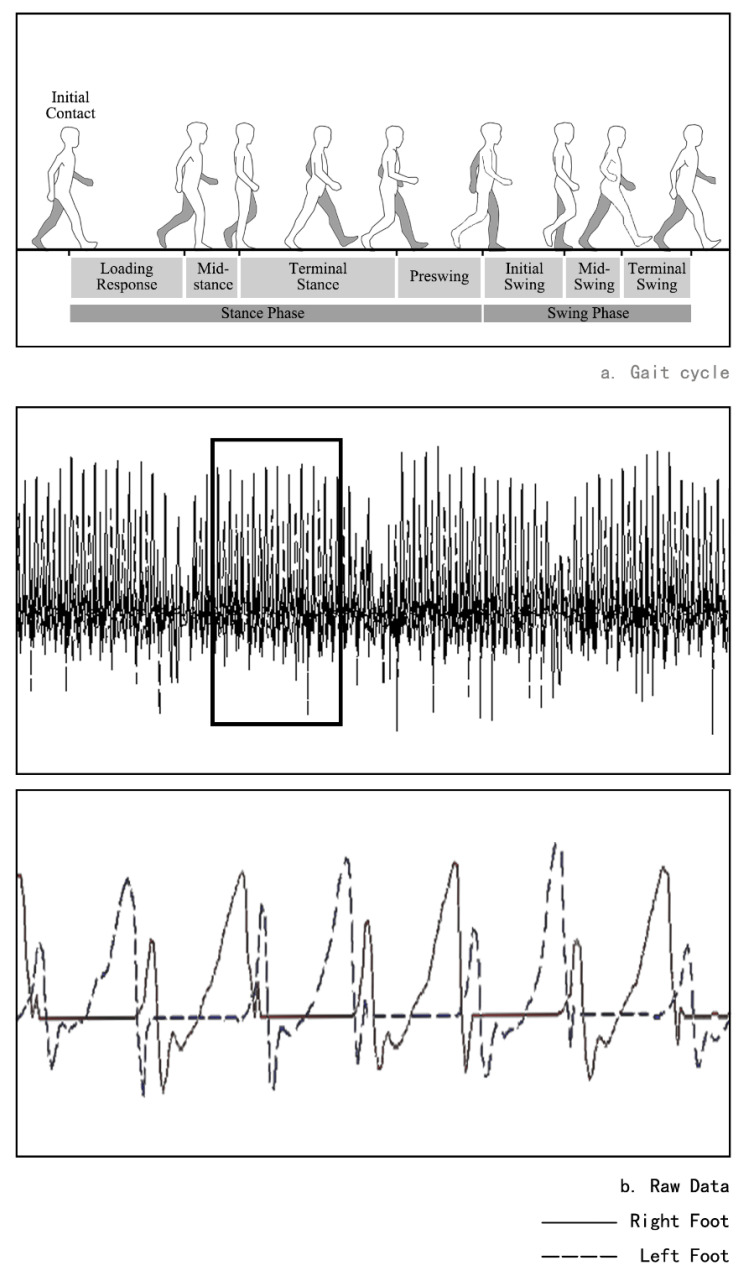
Gait cycle and raw data. (**a**) Schematic diagram of the gait cycle; (**b**) schematic diagram of raw data collected by IDEE.

The following gait variables were evaluated: (1) Walking speed (m/s): the distance traveled along the runway per unit time. (2) Stride length (m): the distance from one heel to the same heel touching the ground again during walking. (3) Step frequency (steps/min): the number of steps per minute. (4) Single support time/double support time (%): single support time refers to the time spent using monopod support in a gait cycle, reflecting the stability of the subjects during walking; double support time refers to the time taken by the use of biped support in a gait cycle, and the increase of its value indicates the weakening of the stability of the subjects during walking. Single support time/double support time reflects the stability of the subjects when walking, and the higher the ratio, the better the stability of the subjects. The division of the gait cycle is shown in Figure 4a. (5) Swing work (g/1 g = 9.8 m/$s^{2}$): at the end of the swing phase, the average longitudinal acceleration of the foot is defined as the thigh swing work, and the swing work represents the strength of the process. (6) Leg falling strength (g/1 g = 9.8 m/$s^{2}$): the average acceleration of a small period of acceleration at the end of the swing phase. The test results are represented by the mean ± standard deviation (mean ± SD).

### 2.5. Statistical Analysis and K-Nearest Neighbor (KNN) Classification Algorithm

After the data acquisition was completed, the data was saved in the main recorder and downloaded to the computer, and IDEEA Version 3.01 (IDEEA3, MiniSun, Fresno, CA, USA) was used for analysis [34]. The raw data are shown in Figure 4b, the software equipped with the equipment can be used to intercept the range of gait data needed and process and analyze the gait data for a specific period of time.

Statistical analysis was carried out with SPSS 21.0 (SPSS, Chicago, IL, USA) statistical software package. Shapiro–Wilk normality test was used to test the normality of data. A paired sample t-test was used to compare the differences of gait data without standing intervention and standing on two different hardness planes for 4 h. An independent sample t-test was used to analyze the differences between males and females. *p* < 0.05 indicates that the difference was statistically significant.

**Figure 5:**
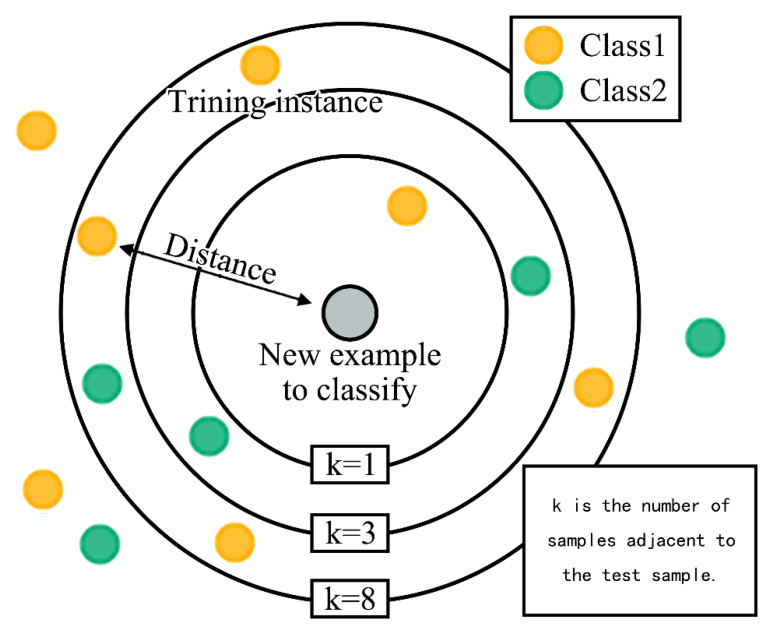
Example of KNN classification.

The K-nearest neighbor (KNN) classification algorithm is a low complexity and theoretically mature data mining algorithm. Its basic idea is: in a sample space if the k other samples closest to a sample belong to the same class, then the sample also belongs to the same class [47]. As shown in Figure 5, the gray test circle sample could have been classified as the first class of the yellow circle or the second class of the green circle. When the number of adjacent samples is 1, k = 1, it is assigned to class 1. When the sample number of the nearest neighbor is 3, k = 3, it is divided into class 2 because there are two green circles and one yellow circle in the rings of k = 3. When the number of adjacent samples is 8, it is divided into class 1 because there are five yellow circles and three green circles in the rings of k = 8. Therefore, using different k values may produce different classification results, the classification will also be affected by different parameters. In this study, we developed a KNN classification algorithm program based on Python. We combined it with “leaving one cross-test” to train and test the prediction accuracy when using different parameters and k values (range 3–8). The algorithm randomly selected 80% of the sample size of the data set as the training set and 20% of the sample size as the test set. After training the algorithm, the correct rate of gait parameter prediction of non-fatigued and fatigued state was tested after standing on the mat for 4 h.

## 3. Results

**Figure 6:**
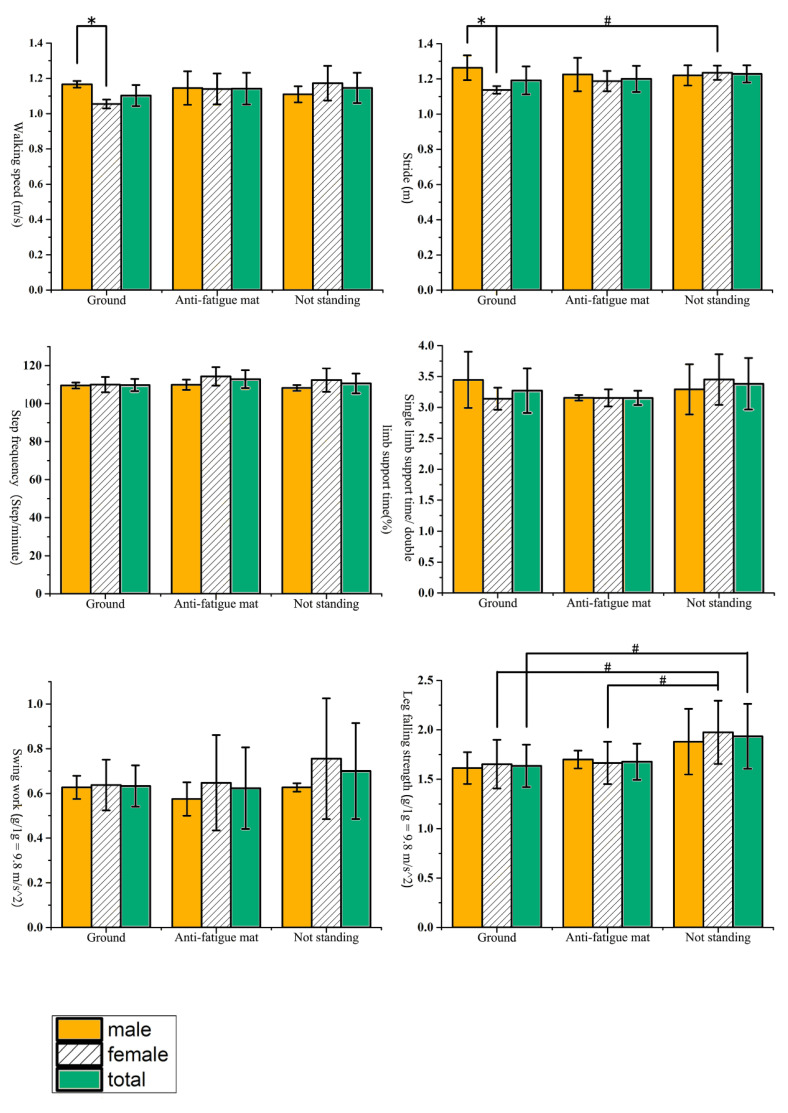
Gait parameters after standing for 4 h on surfaces with different hardness and gender. *, Under the same intervention conditions, there are significant differences in this index between females and males. #, Under the condition of the same sex, there was a significant difference in this index between standing for 4 h and not tired.

According to the Shapiro–Wilk test, the gait data of the subjects followed a normal distribution (*p* > 0.05). The comparison of gait parameters between subjects standing on the ground and anti-fatigue mats for 4 h and before standing are shown in Table 2 and Figure 6. The scale of subjects’ subjective feelings is shown in Table 3.

**Figure 7:**
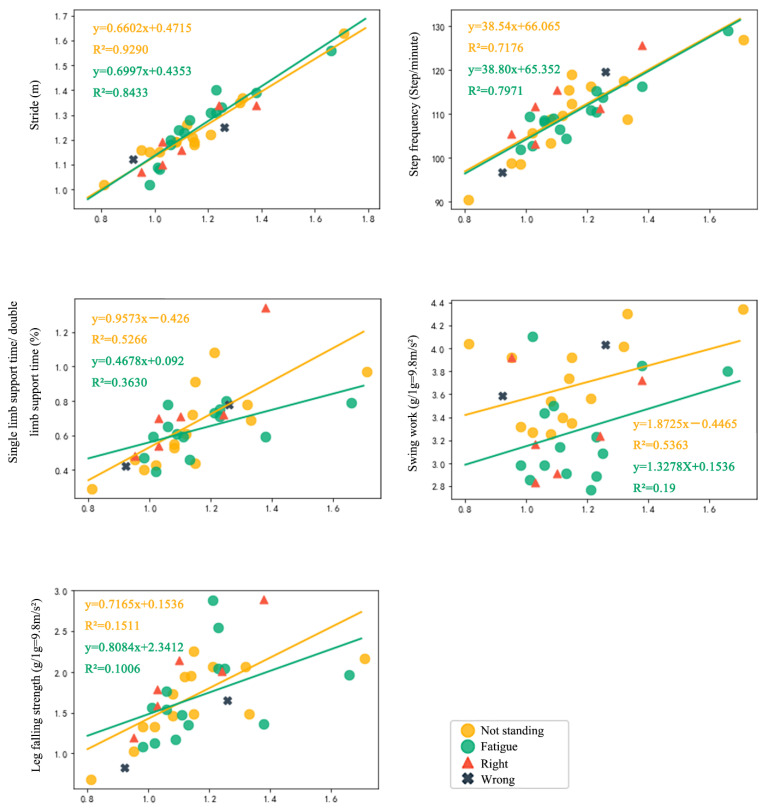
Superposition of walking speed and other gait parameters to fit the linear regression and the accuracy of training.

The data of different indexes and k values on the prediction accuracy are shown in Table 4. In this study, the k value was set to 4 and was selected for gender, height, weight, walking speed, stride length, step frequency, single support time/double support time, swing work, and leg falling strength. In addition, Euclidean distance was used as the distance measure in the study, and the reciprocal of distance was used as the weight of assigned variables. Figure 7 shows the best-fitting linear regression line and the scatter plot of the data distribution of the superposition of the subject’s speed and other gait parameters and marks the correct and wrong data points predicted by the KNN algorithm.

### 3.1. Walking Speed

The subjects’ walking speed after standing on the ground for 4 h, standing on the anti-fatigue mat for 4 h, and without standing intervention is shown in Figure 6, reflecting the distance the subjects walked along the runway in unit time. The faster the walking speed, the higher the subjects’ ability to move. In this study, regardless of gender differences, subjects’ walking speed on the ground for 4 h was lower than that after standing on mats for 4 h and was lower than that without fatigue. Still, the results were not statistically significant (*p* > 0.05). The subjects were divided into groups according to sex. The walking speed of males after standing on the ground for 4 h was significantly faster than that of females (*p* < 0.05), but there was no difference between males and females after standing on mats for 4 h and without standing intervention (*p* > 0.05). Under the condition of the same sex, there was no significant difference in walking speed between males and females after standing on the ground for 4 h, standing on anti-fatigue mats for 4 h, and without standing intervention (*p* > 0.05).

### 3.2. Stride Length

The subjects’ stride length after standing on the ground for 4 h, standing on an anti-fatigue mat for 4 h, and without standing intervention is shown in Figure 6, reflecting the distance from one heel to the same heel touching the ground again during walking. For the same person, the larger the stride length, the better the exercise ability. In this study, regardless of gender differences, the subjects had the largest stride length without fatigue and the most diminutive stride length after standing on the ground for 4 h. Still, the results had no significant difference (*p* > 0.05). The subjects were divided into groups according to sex. The stride length of male subjects after standing on the ground for 4 h was significantly larger than that of females (*p* < 0.05), but there was no difference between males and females after standing on mats for 4 h and without standing intervention (*p* > 0.05). Under the condition of the same sex, the stride length of females after standing on the ground for 4 h was significantly lower than that without standing intervention (*p* < 0.05), but no significant difference was observed between standing on the mat for 4 h and without standing intervention (*p* > 0.05). No significant differences were observed in males (*p* > 0.05).

### 3.3. Step Frequency

The step frequency of the subjects after standing on the ground for 4 h, standing on the anti-fatigue mat for 4 h, and without standing intervention is shown in Figure 6. The step frequency reflects the number of steps the subjects walk in a unit time, and the step frequency and step length determine the walking speed. In general, when the walking speed and step length are constant, the faster the step frequency is, the stronger the subjects’ actionability is. In this study, regardless of gender differences, the subjects’ walking frequency was the fastest after standing on the mat for 4 h and the slowest after standing on the ground for 4 h. Still, there was no significant difference (*p* > 0.05).

### 3.4. Single Support Time/Double Support Time

The single support time/double support time of the subjects after standing on the ground for 4 h, standing on the anti-fatigue mat for 4 h, and without standing intervention is shown in Figure 6, reflecting the stability of the subjects during walking. The greater the percentage of single support time to gait cycle, the greater the single support time/double support time, and the higher the value, the better the stability of walking. In this study, regardless of gender differences, the single support time/double support time was the largest when the subjects were not tired, and the minimum was 4 h after standing on the mat. Still, there was no statistically significant difference (*p* > 0.05).

### 3.5. Swing Work

The swing work of the subjects after standing on the ground for 4 h, standing on the anti-fatigue mat for 4 h, and without standing intervention is shown in Figure 6, which reflects the strength of the swinging legs while walking. The greater the swing work, the more muscular the muscle strength of the lower limbs. In this study, regardless of gender differences, the subjects had the highest swing work without fatigue and the lowest swing work after standing on the mat for 4 h. Still, there was no significant difference between the two groups (*p* > 0.05).

### 3.6. Leg Falling Strength

The leg falling strength of the subjects after standing on the ground for 4 h, standing on the anti-fatigue mat for 4 h, and without standing intervention were shown in Figure 6, which reflects the acceleration of the subjects at the end of the leg swing, and the higher the value, the stronger the muscles of the lower limbs. In this study, without considering the gender difference, the leg falling strength of the subjects was the highest when they were not tired, and the leg falling strength was the lowest after standing on the ground for 4 h. There was a significant difference compared with that without standing intervention (*p* < 0.05). After standing on the mat for 4 h, the leg falling strength was lower than that without standing intervention, but there was no significant difference (*p* > 0.05). When the subjects were divided into groups according to sex, the leg fall intensity of female subjects standing on the ground for 4 h was significantly lower than that without standing intervention (*p* < 0.05), but there was no significant difference between mat and male subjects (*p* > 0.05).

### 3.7. Subject Subjective Feelings

Table 4 describes the mean and standard deviation of subjects’ perception of discomfort in different body parts after standing on different surfaces. When standing on hard ground, subjects rated fatigue and discomfort in the feet, calves, knees, and lower back higher. The subjects rated knee and lower back fatigue and discomfort higher while standing on the floor mat.

### 3.8. K-Nearest Neighbor (KNN) Classification Algorithm

The accuracy of the prediction using different parameters and k values is shown in Table 4. The results show that when the demographic and gait parameters are used to predict standing fatigue, and the k value is 4, the prediction of standing fatigue can reach a moderate accuracy (75%). Figure 7 shows the best fit linear regression line of the superposition of walking speed and other gait parameters and provides examples of the point distribution of training sets and prediction sets and testing right and wrong points. Using the trained algorithm to test the gait parameters after standing on the mat for 4 h, the accuracy of the fatigue state was 38.9%, and the correct rate of the state without intervention was 44.4%.

## 4. Discussion

Muscle discomfort and fatigue caused by standing for a long time can lead to permanent illness [2,16] and affect gait [48]. Workers in these professions often stand for hours without specially designed safeguards, which can lead to falls or other injuries, and females may be more severely affected. Therefore, it is necessary to take intervention measures to reduce related hazards and risks and monitor standing fatigue.

A recent study reviewed the results of the anti-fatigue mat with mixed results [8]. Only medium-level evidence supports that the use of soft materials can alleviate the physical discomfort caused by standing for a long time, and it is impossible to make a conclusive assessment of the effect of the intervention. In addition, the review report said that some studies have found that gender differences impact this discomfort and fatigue. However, there is still not enough evidence, so more research is needed.

The purpose of this study was to observe whether the use of anti-fatigue mats can alleviate lower limb fatigue and discomfort caused by standing for a long time and try to predict standing fatigue by gait parameters. For this reason, we studied the effect of lower limb fatigue on gait characteristics after 4 h of continuous standing work (10-min intervals). Most subjects reported physical solid discomfort and fatigue when standing on hard ground, including soreness in the legs and pain in the soles of their feet, which were much improved when using mats. In addition, it is worth noting that some subjects reported knee and lower back discomfort after standing on the mat for four hours. This may be because the soft surface causes the foot to be lower than usual, resulting in a change in joint position during standing. However, this phenomenon cannot be further analyzed with the available evidence, and this is not the purpose of this study, which will be investigated in the future. Therefore, we assume that the discomfort and fatigue caused by standing for 4 h will significantly impact gait characteristics. The use of anti-fatigue mats will weaken this effect, and there are differences between different genders.

We found that when gender was not taken into account, the intensity of leg falling after standing on the ground for 4 h was significantly lower than that without standing intervention (*p* < 0.05). This difference was not observed when using anti-fatigue mats. This may indicate that the muscle strength of the lower extremities decreases significantly after standing on the ground for a long time, which may be due to the influence of discomfort and fatigue on the joints and muscles of the lower extremities [49]. The study of Zhihui Liu et al. seems to prove this point. In this study, the subjects stood for a long time on planes with different degrees of tilt, and the results of surface EMG showed that the muscles of lower limbs and waist of the subjects appeared a certain degree of fatigue after the intervention [50]. The other five gait parameters showed no significant difference (*p* > 0.05), and no difference was found between using and not using an anti-fatigue mat (*p* > 0.05). According to sex, we found that the stride length of female subjects decreased significantly after standing on the ground for 4 h (*p* < 0.05), and the stride length and stride speed of female subjects were significantly lower than those of males after standing on the ground for 4 h (*p* < 0.05). This phenomenon was not found when anti-fatigue mats were used. This may indicate that females are more vulnerable to discomfort and fatigue caused by standing for a long time and that pain or fatigue adversely affects exercise ability, resulting in a decrease in stride length and speed. This suggests that the use of anti-fatigue mats can reduce this adverse effect. Previous studies have found similar results. Garcia et al. [7] reported significant muscle fatigue effects after 30 min of standing. King et al. [4] also observed discomfort and fatigue caused by prolonged standing, which could be reduced by the use of anti-fatigue cushions or insoles. This was also illustrated by the subjective feedback of the subjects, most of whom experienced pain and fatigue in the soles of their feet, calves, thighs, and back after standing on the ground for four hours, while this discomfort decreased when using mats. Conversely, in a study of gait characteristics of the elderly [51], Seung-uk Ko reported that gait speed, frequency, and stride length all decreased significantly after exercise, while our results showed that subjects’ gait frequency did not change significantly before and after fatigue, which may be because gait performance decreased with age after exercise fatigue, and similar results were obtained in Seung-uk Ko’s study. In Urs Granacher’s study [52], the stride length of young subjects also decreased significantly after fatigue intervention. In this study, subjects fatigue of quadriceps femoris caused by repeated constant knee extension exercise, which may lead to a decrease in thrust during walking, resulting in a shorter stride length, which may be different from the mechanism of fatigue caused by standing for a long time, and its effect on gait characteristics may be different.

When walking, muscle strength, proprioception, and balance ability are all very important to completing walking movements, and muscle fatigue and physical discomfort will negatively impact the movements. Therefore, it is theoretically feasible to predict fatigue by gait parameters, and some studies have proven that there is a correlation between gait parameters and fatigue [36,37,38,39]. The results of the KNN classification algorithm show that the prediction of standing fatigued by demographic parameters and gait parameters is moderately accurate (75%), which indicates that fatigue can be predicted to some extent by monitoring gait parameters. In addition, the matching degree of gait parameters to standing fatigue and a non-fatigued state after standing 4 h on the mat is relatively low. Still, the correct rate of matching non-fatigued states (44.4%) is higher than that of matching fatigued states (38.9%). This may indicate that the gait characteristics of subjects standing on mats for 4 h are between fatigued and non-fatigued. Still, the degree of fatigue is lower than that of standing on the ground for 4 h [53,54].

At present, most studies use subjective evaluation, static plantar pressure, and EMG to evaluate the effect of the anti-fatigue mat. In contrast, in this study, a portable gait analyzer was used to evaluate the function of the lower limbs after standing for a long time and try to use gait parameters to achieve the accurate detection of standing fatigue. However, this study also has limitations, such as the inability to measure more gait parameters, such as joint angle, due to equipment limitations. There were only 18 healthy young subjects, and the number of samples was limited, which reduced the information value of this study. The low sample size will also have an impact on the accuracy of machine learning algorithms. The research sample will be expanded in the future, more indicators will be selected, and more evidence will be provided to verify the research results. In addition, in this study, the subjects only received standing intervention for 4 h, and the subjects may not have reached a high level of fatigue.

## 5. Conclusions

This study compared the gait parameters and proprioceptive sensation of subjects standing on different hardness surfaces for 4 h, respectively. The KNN classification algorithm was used to predict standing fatigue through gait parameters. The following results were obtained: (1) In male subjects, no differences were observed in gait parameters between standing for a long time and without standing intervention, indicating that standing for a long time may not significantly affect the gait characteristics of males. (2) Among the male subjects, using an anti-fatigue mat showed no effect on gait characteristics after standing for a long time, which could be interpreted as high control ability and endurance ability compared with the female group. (3) Among the female subjects, it was observed that the stride length and leg strength after standing on the ground for 4 h were significantly lower than those without standing intervention. Even though, the differences were not observed after using an anti-fatigue mat. Therefore, the use of anti-fatigue mats may reduce the discomfort and fatigue caused by standing for a long time in the female group. (4) After standing on the ground for 4 h, males’ stride length and walking speed were significantly higher than females. While this phenomenon was not observed in anti-fatigue mats, which indicated that females were less resistant to the harmful effects of standing for a long time than males. The use of anti-fatigue mats may improve females’ fatigue and discomfort after standing for a long time. (5) Most subjects reported muscular discomfort and fatigue of their feet, shanks, thighs, and back after standing on the ground for 4 h, while the discomfort experience decreased when using anti-fatigue mats, the soft surface of the anti-fatigue mats may make standing more comfortable. (6) The results of using demographic and gait parameters to predict standing fatigue show moderate accuracy, which indicates that this method was reliable. Gait parameters were similar between those in a non-fatigue state and standing on soft a mat for 4 h, which indicated that an anti-fatigue mat could reduce the negative effect caused by standing for a long time.

## Figures and Tables

**Table 1:** Basic characteristics of the subjects.

N	Age (year)	Weight (kg)	Height (m)	Female/Male
18	23.2 ± 1.6	61.1 ± 8.0	1.68 ± 0.06	10/8

**Table 2:** Gait parameters after standing for 4 h on surfaces with different hardness and gender.

Indexes (Unit)	Ground			Anti-Fatigue Mat			Before Standing		
	Male	Female	Average	Male	Female	Average	Male	Female	Average
Walking speed (m/s)	1.17 ± 0.02	1.05 ± 0.03 *	1.10 ± 0.06	1.14 ± 0.10	1.14 ± 0.09	1.14 ± 0.09	1.11 ± 0.05	1.17 ± 0.10	1.15 ± 0.09
Stride length (m)	1.26 ± 0.07	1.14 ± 0.02 *#	1.19 ± 0.08	1.23 ± 0.10	1.19 ± 0.06	1.20 ± 0.07	1.22 ± 0.06	1.24 ± 0.04	1.23 ± 0.05
Step frequency (step/minute)	109.51 ± 1.60	109.98 ± 4.03	109.78 ± 3.23	109.94 ± 2.70	114.33 ± 4.83	112.86 ± 4.72	108.23 ± 1.56	112.40 ± 6.24	110.61 ± 5.25
Single support time/double support time (%)	3.45 ± 0.45	3.14 ± 1.78	3.27 ± 0.36	3.15 ± 0.04	3.15 ± 0.14	3.15 ± 0.12	3.29 ± 0.41	3.45 ± 0.41	3.38 ± 0.42
Swing work (g/1 g = 9.8 $m/s^{2}$)	0.63 ± 0.05	0.64 ± 0.11	0.63 ± 0.09	0.57 ± 0.07	0.65 ± 0.21	0.62 ± 0.18	0.63 ± 0.02	0.76 ± 0.27	0.70 ± 0.21
Leg falling strength (g/1 g = 9.8 $m/s^{2})$	1.61 ± 0.16	1.65 ± 0.25 #	1.64 ± 0.21 #	1.70 ± 0.09	1.66 ± 0.21 #	1.68 ± 0.18	1.88 ± 0.33	1.97 ± 0.32	1.93 ± 0.33

*, Under the same intervention conditions, there are significant differences in this index between females and males. #, Under the condition of the same sex, there was a significant difference in this index between standing for 4 h and not tired.

**Table 3:** Subjective feeling rating scale.

	Foot	Ankle	Lower Leg	Knee	Upper Leg	Hip	Lower Back
Ground	4.28 ± 0.73	2.67 ± 0.75	2.83 ± 0.76	3.28 ± 0.87	2.17 ± 0.90	2.61 ± 0.89	2.61 ± 0.76
Mat	2.39 ± 0.59	2.22 ± 0.53	2.50 ± 0.76	2.94 ± 1.08	2.06 ± 0.91	2.28 ± 0.73	2.56 ± 1.01

Responses based on scale of 1 through 5 (1 = none, 2 = little, 3 = some, 4 = quite a bit, 5 = very much).

**Table 4:** Prediction accuracy of KNN classification algorithm with different parameters and k values.

k-Value	Parameter Selection		
	Gender, Height, Weight, Walking Speed, Stride Length, Step Frequency, Single Support Time/Double Support Time, Swing Work, and Leg Falling Strength	Walking Speed, Stride Length, Step Frequency, Single Support Time/Double Support Time, Swing Work, and Leg Falling Strength	Walking Speed, Stride Length, Step Frequency, and Single Support Time/Double Support Time
k = 3	25.0%	25.0%	25.0%
k = 4	75.0%	50.0%	50.0%
k = 5	50.0%	37.5%	37.5%
k = 6	62.5%	25.0%	25.0%
k = 7	62.5%	25.0%	25.0%
k = 8	50.0%	12.5%	12.5%

k is the number of samples adjacent to the test sample.

## Data Availability

The data that support the findings of this study are available on reasonable request from the corresponding author. The data are not publicly available due to privacy or ethical restrictions.
